# Serological Blood Group Discrepancy and Cold Agglutinin Autoimmune Hemolytic Anemia Associated With Novel Coronavirus

**DOI:** 10.7759/cureus.11495

**Published:** 2020-11-15

**Authors:** Babita Raghuwanshi

**Affiliations:** 1 Pathology- Transfusion Medicine, All India Institute of Medical Sciences, Bhopal, IND

**Keywords:** cold agglutinin, novel coronavirus, autoimmune haemolytic anemia

## Abstract

A case of a 45-year-old male presentation with viral pneumonia with anemia and thrombocytopenia and was admitted to COVID-19 ICU. The blood bank encountered a discrepancy in blood grouping and cross-match, which were subsequently resolved. The patient presented to the emergency room with fever and shortness of breath. He had tachycardia, fever, and had an oxygen saturation of 88% on room air. His SARS-CoV-2 RT-PCR test was positive. Total and unconjugated bilirubin was raised. Due to the anemia and falling haemoglobin, he was not started on any anticoagulation. On investigation, the patient's direct Antiglobulin test was positive. Cold agglutinin titer at 4-degree Celsius was 64. The blood sample showed auto agglutination at collection and discrepancy in blood grouping and cross-matching, which were subsequently resolved. As observed in this case report, COVID-19 infection can be associated with cold agglutinin disease and Autoimmune hemolytic anemia, and cold agglutinins should be recognized as potentially significant due to interference with laboratory investigations and complications associated with COVID 19.

## Introduction

COVID-19 causes a disease with symptoms ranging from asymptomatic, mild to severe disease. We report a case of serological blood group discrepancy and cold agglutinin autoimmune hemolytic anemia associated with the novel coronavirus. There are two types of cold agglutinin disease (CAD). The Primary CAD is idiopathic and secondary CAD is associated with autoimmune disorders and viral infections. In CAD, agglutination of red blood cells occurs at below normal body temperature and is associated with extravascular hemolysis leading to hemolytic anemia [1].

## Case presentation

A 45-year-old male was admitted to COVID-19 ICU with RT-PCR positive for COVID-19. The blood bank received a request for two units of packed red cells for the patient along with the patient's sample. The sample showed autoagglutination when received at the blood bank. The patient had no history of blood transfusion in the past.

The initial haemoglobin of the patient was 6.9g/dl, and C-reactive protein (CRP) was raised to 120.10mg/dl. There was a noticeable increase in CRP and a drop in Hb to 6.0g/dl coinciding with the worsening of pneumonia. Total leukocyte count was 5.98 X10 3/ microliter and platelet count was 30X 10 3 / Microliter. His course was further complicated by progressive thrombocytopenia, with a nadir of a platelet count of 20 X 10 3 / microliter. Reticulocyte count was 1.15%. Urea was elevated to 105.34mg/dl and creatinine to 2.01mg/dl. He developed renal failure and renal replacement therapy was advised. Alkaline phosphatase was 310.21U/L. Serum bilirubin was elevated to 7.76mg/dl, and unconjugated bilirubin was 2.91 mg/dl. Total protein was low (4.24g/L), Albumin was 2.03g/L. The presence of target cells and spherocytes in blood films was observed indicative of autoimmune hemolytic anemia (AIHA) [2]. The patient's sample was received in the blood bank for blood grouping and cross-matching. The results of cell and serum grouping showed ABO discrepancy (Table 1).

**Table 1:** Initial ABO Discrepancy

Anti A	Anti B	Anti AB	Anti D	A1 cell	B Cell	O Cell	Auto control
4 +	4 +	4 +	4 +	4 +	2 +	2 +	4 +

The direct Coombs test was positive. Reverse grouping at 4 degrees Celcius showed increased agglutination of B and O cells, suggesting the presence of cold agglutinins. This antibody reacted with all patient and donor RBCs at cold temperatures. Both the Ethylenediaminetetraacetic acid (EDTA) tube and plane tube were kept at 37 degrees Celcius. Prewarmed saline kept at 37 degrees Celcius was used to wash one ml of red cells of the patient. Cell grouping was done with red cells washed with prewarmed saline in prewarmed tubes with anti-A, Anti B, and Anti AB. A 5% red cell suspension of A, B, and O cells were made and kept at 37 degrees. Patient serum was separated and kept at 37 degrees centigrade for 1 hr. An equal volume of serum and Red cells were incubated at 37 degrees for 45 minutes. The auto-adsorbed serum was used for cross-matching. A Prewarmed pipette tip was used for cell and serum grouping and cross-matching. The ABO Discrepancy was resolved with a warm saline wash of patient red cells and the use of prewarmed serum for serum grouping, and the results are elaborated in Table 2.

**Table 2:** ABO Discrepancy resolved after a prewarming technique.

Anti A	Anti B	Anti AB	Anti D	A1 cell	B Cell	O Cell	Auto control
0	4 +	4 +	4 +	4 +	0	0	2 +

The direct Coombs test was positive. The blood group was Interpretated as B Positive. The patient had no history of previous transfusions therefore previous exposure to blood products and the presence of Red cell alloantibody was unlikely. Rh typing of the patient cell was done using the prewarming technique (Table 3).

**Table 3:** Rh Phenotype of the patient.

Anti D	Anti C	Anti c	Anti E	Anti e
+	+	+	0	+

Crossmatch was performed using auto-adsorbed prewarmed serum by Tulip gel cards, and Rh matched compatible units were selected for the patient. Blood grouping was done by tube technique and extended Rh phenotype for ANTI-C, ANTI-c, ANTI-E, ANTI-e was done by Tulip reagents. A cold agglutinin titer was performed using pooled O Cells from donors, and phosphate buffer saline and patient serum separated and kept at 37 degrees Celcius. Serial dilution was prepared, and titer was negative at 37 degrees Celcius, the titer of 2 at room temperature, and a titer of 64 at 4 degrees Celcius was observed. This antibody reacted with all patient and donor RBCs at cold temperatures.

Rh compatible units were cross-matched with auto adsorbed serum of the patient, and compatible B Positive units were safely transfused. It was advised to keep the patient warm, monitor the patient during transfusion with a slow transfusion of blood products. The transfusion was uneventful. ABO Discrepancy was resolved after a warm saline wash of patient red cells, and prewarmed serum was used for serum grouping.

## Discussion

The presence of cold agglutinins in COVID 19 patients has been observed in several reports which showed that SARS-CoV-2-infected individuals developed autoimmune hemolytic anemia (AIHA) [2-8]. Nevertheless, the prevalence and implications of such findings remain to be elucidated. The thermal amplitude of cold agglutinins in our study was zero at 37 degrees centigrade and 64 at 4 degrees Celsius and caused ABO Discrepancy and interfered with cross-matching. In our case, the antibody specificity of cold agglutinin could not be determined. However, the cold antibody reacted with the patient's and donor red blood cells (RBCs) at cold temperatures. The management included avoidance of cold exposure, maintaining haemoglobin, and treatment of underlying etiology. The serological blood group and cross match discrepancy observed in our case was resolved using the prewarmed saline wash technique, and the use of auto adsorbed serum. The limiting factor in our study was our inability to document the antibody specificity in cold agglutinin autoimmune hemolytic anemia and the association with other illnesses like atypical pneumonia infection, which may be associated with novel coronavirus.

## Conclusions

The importance of bedside protocols during transfusion in patients with cold agglutinins is emphasized with monitoring of transfusion, keeping the patient warm, and use of blood warmers. More vigilance is required in the care of patients affected with COVID-19, and the increasing importance of a multidisciplinary team approach in management is emphasized as the spectrum of complications secondary to COVID‐19 increases.

## References

[1] Berentsen S, Tjønnfjord GE (2012). Diagnosis and treatment of cold agglutinin mediated autoimmune hemolytic anemia. Blood Rev.

[2] Gehrs BC, Friedberg RC (2002). Autoimmune hemolytic anemia. Am J Hematol.

[3] N. R. Patil, E. S: Herc (2020). M Girgis: Cold agglutinin disease and autoimmune hemolytic anemia with pulmonary embolism as a presentation of COVID-19 infection. Hematol Oncol Stem Cell Ther.

[4] Rosenzweig JD, McThenia SS, Kaicker S (2020). SARS-CoV-2 infection in two pediatric patients with immune cytopenias: a single institution experience during the pandemic. Pediatr Blood Cancer.

[5] Lazarian G, Quinquenel A, Bellal M (2020). Autoimmune haemolytic anaemia associated with COVID-19 infection. Br J Haematol.

[6] Lopez C, Kim J, Pandey A (2020). Simultaneous onset of COVID-19 and autoimmune hemolytic anemia. Br J Haematol.

[7] Zagorski E, Pawar T, Rahimian S (2020). Cold agglutinin autoimmune hemolytic anemia associated with the novel coronavirus (COVID-19). Br J Haematol.

[8] Capes A, Bailly S, Hantson P (2020). COVID-19 infection associated with autoimmune hemolytic anemia. Ann Hematol.

